# Image-based phenotyping for identification of QTL determining fruit shape and size in American cranberry (*Vaccinium macrocarpon* L.)

**DOI:** 10.7717/peerj.5461

**Published:** 2018-08-15

**Authors:** Luis Diaz-Garcia, Giovanny Covarrubias-Pazaran, Brandon Schlautman, Edward Grygleski, Juan Zalapa

**Affiliations:** 1 Instituto Nacional de Investigaciones Forestales, Agrícolas y Pecuarias, Pabellon de Arteaga, Aguascalientes, Mexico; 2 University of Wisconsin-Madison, Madison, WI, USA; 3 Innovation Center, Bayer CropScience NV, Ghent, Belgium; 4 The Land Institute, Salina, KS, USA; 5 Valley Corporation, Tomah, WI, USA; 6 Vegetable Crops Research Unit, USDA-ARS, Madison, WI, USA

**Keywords:** Fruit shape, American cranberry, Persistent homology, Fruit size, QTL mapping, Digital phenotyping

## Abstract

Image-based phenotyping methodologies are powerful tools to determine quality parameters for fruit breeders and processors. The fruit size and shape of American cranberry (*Vaccinium macrocarpon* L.) are particularly important characteristics that determine the harvests’ processing value and potential end-use products (e.g., juice vs. sweetened dried cranberries). However, cranberry fruit size and shape attributes can be difficult and time consuming for breeders and processors to measure, especially when relying on manual measurements and visual ratings. Therefore, in this study, we implemented image-based phenotyping techniques for gathering data regarding basic cranberry fruit parameters such as length, width, length-to-width ratio, and eccentricity. Additionally, we applied a persistent homology algorithm to better characterize complex shape parameters. Using this high-throughput artificial vision approach, we characterized fruit from 351 progeny from a full-sib cranberry population over three field seasons. Using a covariate analysis to maximize the identification of well-supported quantitative trait loci (QTL), we found 252 single QTL in a 3-year period for cranberry fruit size and shape descriptors from which 20% were consistently found in all years. The present study highlights the potential for the identified QTL and the image-based methods to serve as a basis for future explorations of the genetic architecture of fruit size and shape in cranberry and other fruit crops.

## Introduction

The American cranberry (*Vaccinium macrocarpon* L.) is a perennial fruit crop native to North America (Eck, 1990), which has gained importance worldwide because of its reported human health benefits (Neto, 2007; Ermel et al., 2011). Recently, many studies have characterized the cranberry genome, but few studies have studied the genetic architecture governing important horticultural traits. Next-generation sequencing has been used to assemble and study the cranberry plastid (Fajardo et al., 2013), mitochondrial (Fajardo et al., 2014), and nuclear (Polashock et al., 2014) genomes. Similarly, differential gene expression studies during the fruit ripening process have provided information regarding putative genes involved in flavonoid biosynthesis, transport, and regulation (Sun et al., 2015). The recent development of several biparental populations in cranberry (e.g., GRYG and CNJ populations) have allowed the construction of simple sequence repeat based (Georgi et al., 2013; Schlautman et al., 2015b) and single nucleotide polymorphism based genetic maps (Covarrubias-Pazaran et al., 2016; Daverdin et al., 2017; Schlautman et al., 2017).

The growing consumer demand for sweetened dried cranberries (SDC) has increased the need for new cranberry varieties that produce large, round, and uniformly shaped fruit (Cranberry Institute data, https://www.cranberryinstitute.org). The cranberry industry has used fruit sizers to separate large fruit, which are sold at a premium for SDC processing compared with small fruit, which are used for cranberry juices (Nicole Hansen, 2017, personal communication; Cranberry Creek Cranberries, Necedah, WI, USA). Most cranberry breeding programs still use manual caliper measurements to gather data about basic cranberry shape and size attributes, and sometimes use visual assessments to categorize complex shapes of interest.

Basic fruit shape descriptors such as length, width, aspect ratio (i.e., length-to-width (LW) ratio), fruit size, and color have long been measured and used as major determinants of fruit quality in many crops (Monforte et al., 2014). More recently, accessible image-based phenotyping methods have been developed allowing the high-throughput collection of basic fruit quality descriptors. For example, image-based phenotyping and shape modeling methodologies have been used in several crops to capture and analyze fruit shape variation in natural populations as well as in segregating crosses for genetic mapping purposes (Van Eck, Van Der Heijden & Polder, 1998; Perin et al., 2001; Beyer et al., 2002; Gonzalo & Van Der Knaap, 2008; Rodriguez et al., 2011; Tan et al., 2015; Łangowski, Stacey & Østergaard, 2016). When combined with advances in genotyping technologies, these studies have allowed precise identification of numerous loci controlling fruit size and shape in multiple crops (Zhang et al., 2009; Devoghalaere et al., 2012; Walter, Liebisch & Hund, 2015; Wu et al., 2015; Grandillo & Cammareri, 2016). Given their global horticultural importance, the characterization of fruit shape has been especially common in crops from the Solanaceae (Ben Chaim et al., 2001, 2003; Lecomte et al., 2004; Tanksley, 2004; Zygier et al., 2005; Gonzalo & Van Der Knaap, 2008; Martínez-López, Ochoa-Alejo & Martínez, 2014) and Cucurbitaceae families (Monforte et al., 2004; Yuan et al., 2008; Fernández-Silva et al., 2009).

Computer vision methods, such as landmark-based methodologies (Costa et al., 2011) that describe complex shape features in addition to the basic shape descriptors, are widely available. Landmark-based methodologies use strategically located coordinates to mark identifiable features in fruit or leaves, and since the homologous landmarks among different samples can be compared, their use often reveals genetic and developmental features in fruit and leaf shape variation (Chitwood et al., 2016; Li et al., 2017a). Although this methodology is easy to implement, major limitations arise when landmark placing is ambiguous or when obvious homologous features are unavailable for coordinate location. To overcome these limitations, Li et al. (2017a) recently created a landmark-free persistent homology method to numerically quantify the shape features of more than 200,000 leaves representing 141 plant families. Using this methodology, the authors successfully classified leaf shapes into their corresponding family even when shape references (or landmarks) between different leaves were absent.

In this study, we developed a massive phenotyping approach based on digital imaging to rapidly acquire data for basic cranberry fruit shape descriptors such as length, width, and area in breeding programs. We also implemented a newly developed persistent homology methodology to comprehensively quantify complex fruit shape features that are difficult to quantify with traditional descriptors. Moreover, we used data gathered using such computer-vision methodologies in a full-sib mapping population to map genetic regions governing fruit shape and size in cranberry.

## Methods

### Plant material and phenotyping

Phenotypic data was collected for the biparental population GRYG (*n* = 351 individuals), which was previously used in recent cranberry genetic mapping studies (Covarrubias-Pazaran et al., 2016; Schlautman et al., 2017). This population is derived from a cross between the maternal parent [BGx(BLxNL)]95 and the paternal parent GH1x35. During 2014, 2015, and 2016, we harvested all the cranberry fruit within a 0.09 m^2^ quadrat placed in each genotype plot, and randomly selected 25 of those fruit for digital phenotyping using *GiNA* (Diaz-Garcia et al., 2016) in MATLAB. All 25 fruit collected for each genotype were photographed on a white background with six reference circles on each side for size normalization purposes. For each fruit, measurements of length (longest diameter), width (shortest axis, both determined by the centroid of the object), LW ratio, projected area (2D area, as observed from the top) and eccentricity (from 0 to 1, where 0 is a perfect circle and 1 is a line segment) were recorded. In addition, we created individual binary images of all the fruit for the persistent homology analysis described below.

### Shape measurement using persistent homology

In addition to measuring fruit shape using basic attributes such as LW ratio and eccentricity, we applied a topological approach called persistent homology (adapted from Li et al. 2017a, and ran in MATLAB). Briefly, this method assigns a value to each pixel in an image depending on the density of neighboring pixels. To differentiate more complex fruit shapes, this method divides the fruit image into subsets of features using increasing annuli emanating from the center of the shape. The persistent homology algorithm used herein discretized each Euler characteristic curve into 30 values and then concatenated them over four annuli; in this sense, each frxsuit shape was characterized by 120 values. After applying this method, we performed a principal component (PC) analysis on the 120 values measured for each fruit using the function *princomp* in MATLAB. Then, the two first PC1 and PC2 and the other shape descriptors (length, width, LW, area, and eccentricity) were exported to R for further statistical analysis and quantitative trait loci (QTL) mapping. The complete code for image analysis and persistent homology is provided (File S1).

### Statistical analysis

Using image analysis and persistent homology, we measured seven traits, three of them characterizing fruit size (length, width, and projected area), and four characterizing fruit shape/elongation (LW ratio, eccentricity, PC1 and PC2). For each trait on a per-year-basis, we averaged the repeated measurements per genotype (the 25 individual fruit) and proceeded to calculate best linear unbiased predictors (BLUP) by fitting a mixed model *Y*∼*Zu* + ε using the R package *sommer* (Covarrubias-Pazaran, 2016) where *Y* represented the measured trait in a specific year, *Z* was the incidence matrices for random effects (identity matrix of length equals to the number of individuals), and *u* was a variance-covariance matrix (additive relationship matrix *A*). Genomic heritability (*h*^2^) was calculated for each trait within each year according to De Los Campos, Sorensen & Gianola (2015).

### Genetic data and stepwise QTL mapping

The 976 genetic markers genetic used in this QTL mapping study was generated by Covarrubias-Pazaran et al. (2016) and chosen to represent unique locations/recombination bins in the Schlautman et al. (2017) cranberry consensus map. For each year, a four-way *cross* object with seven phenotypes was created in R using the package *qtl* (Broman et al., 2003). QTL detection was performed in the context of a two-dimensional two QTL genome scan using the Haley–Knott regression implemented in the *stepwiseQTL* function. The *stepwiseQTL* function implements a model selection procedure that identifies as many true QTL as possible, while controlling the rate of inclusion of extraneous loci (Manichaikul et al., 2009). We performed QTL mapping using both a univariate (one trait at time) and a covariate approach. The covariate approach was used to partially dissect confounded QTL effects of correlated traits by running *stepwiseQTL* while adding covariates in a complete diallelic fashion. For each trait/year combination, the penalties on the main effects and interactions (*T_m_*, *T_i_^H^* and *T_i_^L^*) were calculated using the function *scantwo* with 1,000 permutations. In total, 21,000 permutations were required for the seven traits evaluated over the 3-year period. To speed up this process, we distributed the permutations among 1,000 jobs performed at the Center for High Throughput Computing of the University of Wisconsin-Madison using the software HTCondor (Thain, Tannenbaum & Livny, 2005) and a custom-made Shell script. At the end, the permutations were automatically collected and the specific penalties for each trait/year combination were calculated and used for selecting significant QTL at α = 0.05. QTL detection using the function *stepwiseQTL* was parallelized using the R package *snow* on a local server. Circos figures were generated in the R package *SOFIA* (Diaz-Garcia et al., 2017) to allow visual inspection of QTL identified and their collocalization across linkage groups.

## Results

### Persistent homology and correlation with other basic descriptors

**Figure 1:**
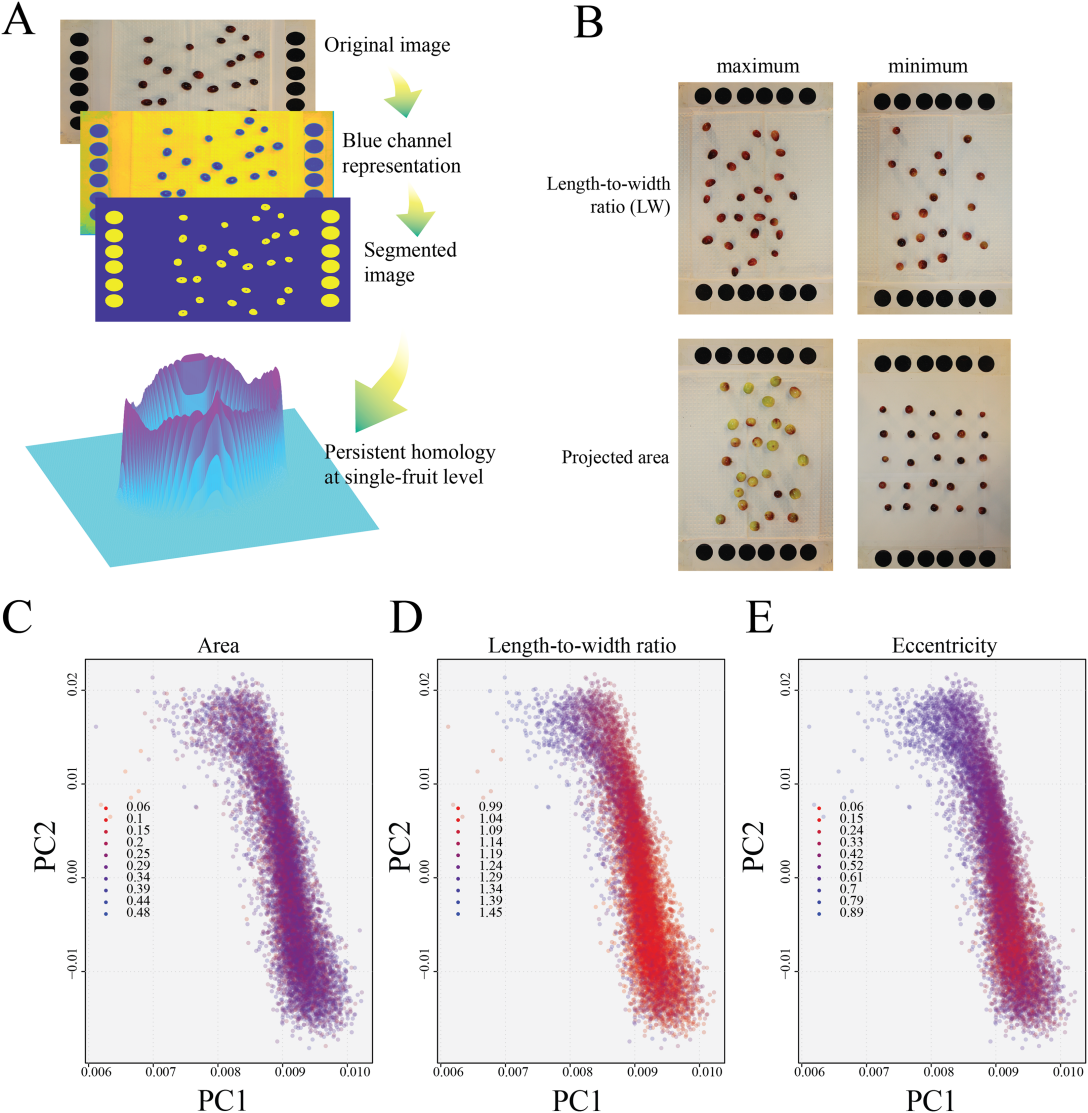
Image processing and persistent homology cranberry fruit characterization. (A) Image processing pipeline: using a conventional digital single-lens reflex (DSLR) camera; images were acquired and then segmented for detection of individual fruit, which were further analyzed to acquire basic descriptors in GiNA, and to perform persistent homology analysis. (B) Genotypes with extreme values for eccentricity and projected area (from the 2014 field season). Comparison of persistent homology PC1 and PC2 with (C) area, (D) length-to-width ratio, and (E) eccentricity, using data from 2014 as an example.

In total, we analyzed 1,053 pictures from 351 full-sib individuals taken over a 3-year period; each picture contained at least 25 cranberry fruit (Fig. 1A). Measurement of fruit basic descriptors (length, width, LW ratio, area, and eccentricity) produced a massive amount of phenotypic data (more than 30,000 fruit phenotyped). Visual inspection of extreme values among these traits revealed highly consistent gathered data; in Fig. 1B, genotypes with the most extreme values for eccentricity and projected area (from the 2014 replicate) are shown as example. After computing basic fruit descriptors with GiNA (Diaz-Garcia et al., 2016), we measured the shape of each fruit using a persistent homology method (Li et al., 2017a). To modify the sensitivity of the persistent homology algorithm, multiple parameters can be adjusted such as increasing/decreasing both the number of blocking annuli and the elements in which each Euler characteristic is decomposed. Because an increment in these two variables considerably multiply the computational resources required, and considering the simplicity of cranberry fruit shapes, we opted to use only four annuli composed of 30 elements.

As expected, the basic attributes determining cranberry fruit size, such as projected area, showed no correlation with persistent homology-based PC1 and PC2 (not average per genotype but single measurements per fruit) (Fig. 1C); however, the basic descriptors characterizing fruit shape (LW and eccentricity) showed a clear correlation with PC1 and PC2 (Figs. 1D and 1E).

**Figure 2:**
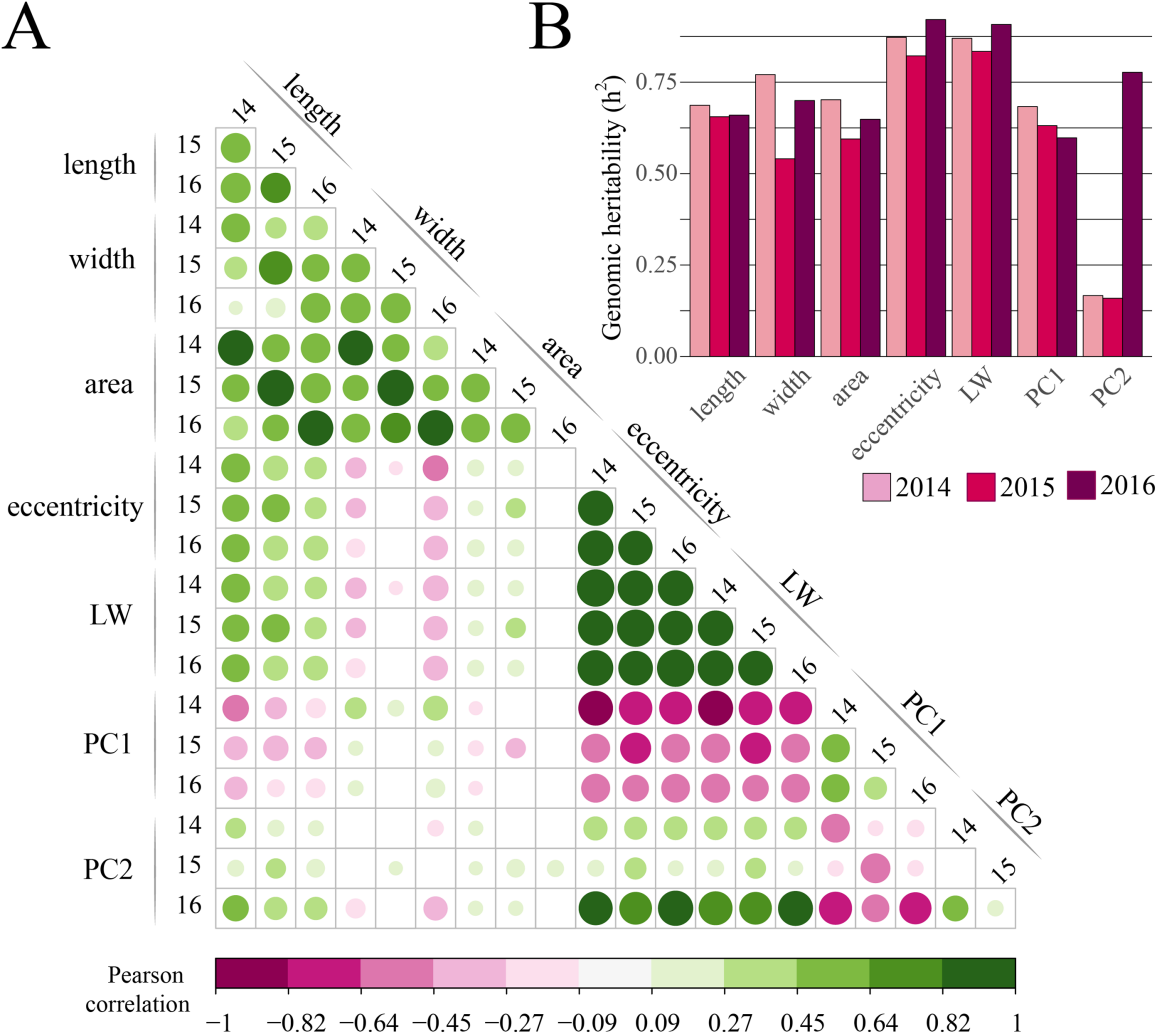
Correlation among cranberry traits and heritability estimates. (A) Pearson correlation between all persistent homology-based and basic descriptors; both circle size and color illustrate the correlation between pairs of traits; circles were omitted for non-significant correlation (at *p* = 0.05); BLUP were used for calculating correlations. (B) Genomic heritability (*h*^2^) by trait calculated on a per-year-basis using genomic relationships between individuals.

Pairwise correlations were computed for the genotype BLUP for each of the seven traits (two persistent homology-based PC and five basic descriptors) on a per-year basis. As expected, high correlations were observed among shape-related (LW, eccentricity, PC1, and PC2) and size-related (length, width, and area) parameters (Fig. 2A). In particular, fruit area was highly correlated with length and width within years (Pearson’s correlation = 0.870–0.951); LW and eccentricity showed a similar trend both within and between years. These two traits showed moderate-to-high negative correlation with PC1 and moderate-to-low negative correlation with PC2. Interestingly, we observed moderate correlation between the PC1 with both eccentricity and LW; however, this was inconsistent among years. We observed low correlation (lower than 0.5) between shape and size related parameters. BLUP for each trait were consistent and correlated among years except for PC2; eccentricity and LW showed correlations among years greater than 0.840.

Estimates for genomic heritability (*h*^2^) on a per-year-basis ranged from 0.159 (PC2, 2015) to 0.921 (eccentricity, 2016) with a mean of 0.676 (Fig. 2B). Averaged across years, eccentricity and LW had the largest *h*^2^ with 0.872 and 0.871, respectively. PC2 had the lowest *h*^2^ with 0.368.

### Stepwise QTL mapping

**Figure 3:**
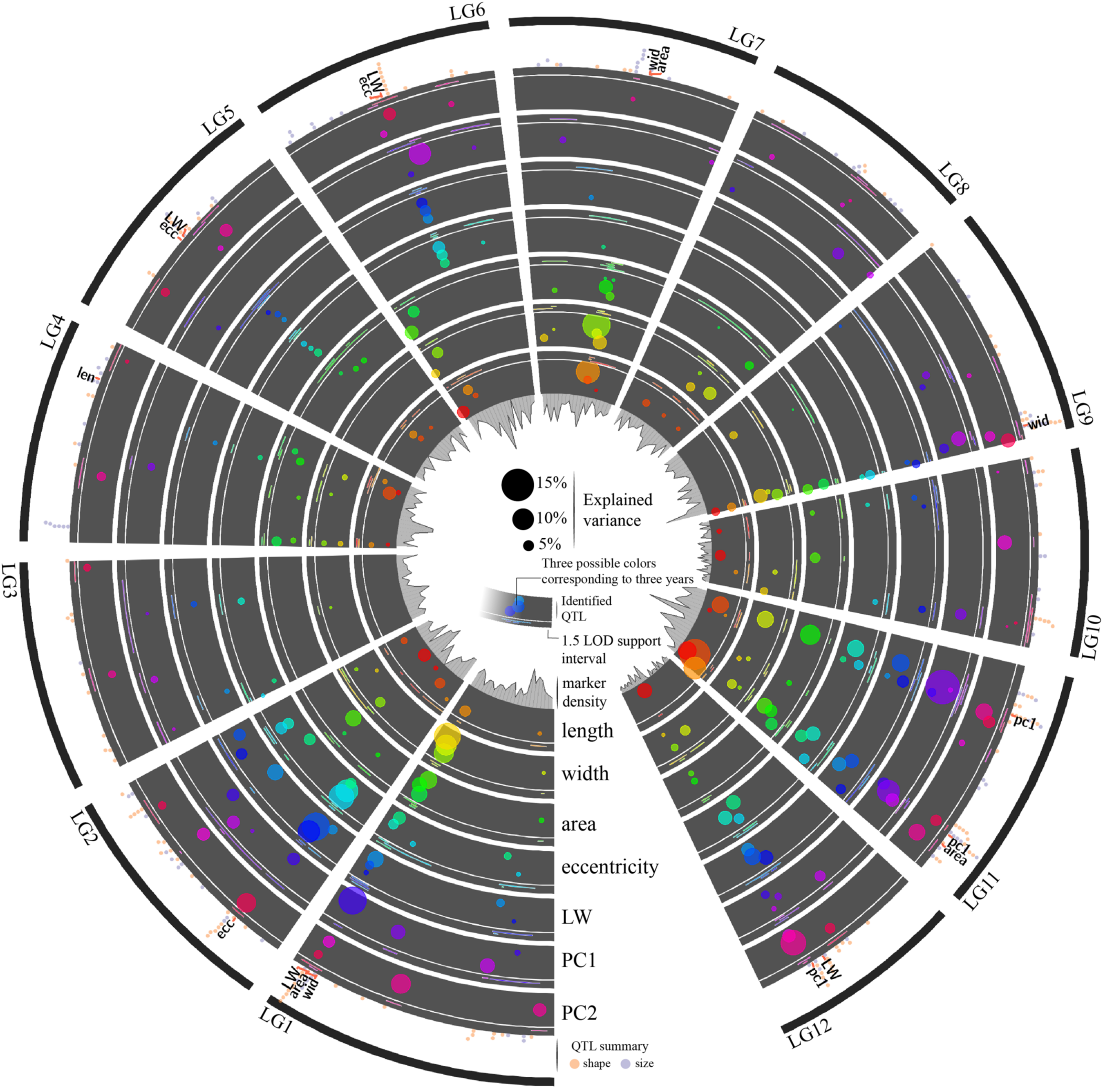
The 252 cranberry QTL identified for fruit shape and size in a 3-year period. QTL are presented as circles within concentric rings for each trait, with the 1.5 LOD support intervals in smaller secondary rings outside each trait track. Circle color scales denote the year each QTL was identified, and circle size is determined by the amount variance explained by the QTL (see scale in the center). The outermost ring summarizes QTL for fruit shape (orange circles) and fruit size related (purple circles) traits to allow visualization of collocalized QTL from multiple traits. Labels in the outer ring denote the 17 QTL identified in all 3 years of evaluation. For specifics about the location and explained variance of each QTL presented here, see File S2.

The methodology used in this study allowed us to identify a large set of well-supported QTL associated with size and shape in cranberry fruit. In total, we identified 252 single QTL for the seven traits and 3 years (Fig. 1 and File S2). The explained variance by QTL models (i.e., fitting all QTL discovered by trait on a given year) ranged from 77.86% (width, 2014) to 87.68% (PC2, 2014). Explained variance by single QTL ranged from 1.13% to 20.89% with a mean and median of 5.80% and 4.99%. After successfully identifying QTL on a per-year-basis, we searched for collocated QTL in all 3 years using an 8 cM overlapping interval as selection criteria. We found 17 different QTL that collocated consistently in the 2014–2016 period (see labels in outer ring of Fig. 3); LW had the most collocated QTL with 4, width, area, eccentricity and PC1 had 3 each, length had 1, and no collocated QTL were found for PC2. QTL found in all 3 years provided additional confidence for those genomic region—trait associations. An additional 30 QTL were found where collocated QTL were present for 2 of the 3 years. In total, consistent QTL in 2 and 3 years added up to 44% of all associations found.

Many of the traits measured in this study were highly correlated (Fig. 2), and we observed multiple collocalizations of QTL among related traits (for fruit size: length, width, and area; for fruit shape: LW, eccentricity, PC1, PC2). Even if they appeared in only 1 or 2 years, collocalization of QTL for related traits suggested that certain genomic regions might have important roles in governing fruit size and shape. For example, there is moderate-strong evidence for a region controlling fruit size at the start of LG4; several QTL (present in up to 2 years) were found in this region for size related traits (Fig. 3, see inner three rings and QTL summary located in the outermost ring).

**Figure 4:**
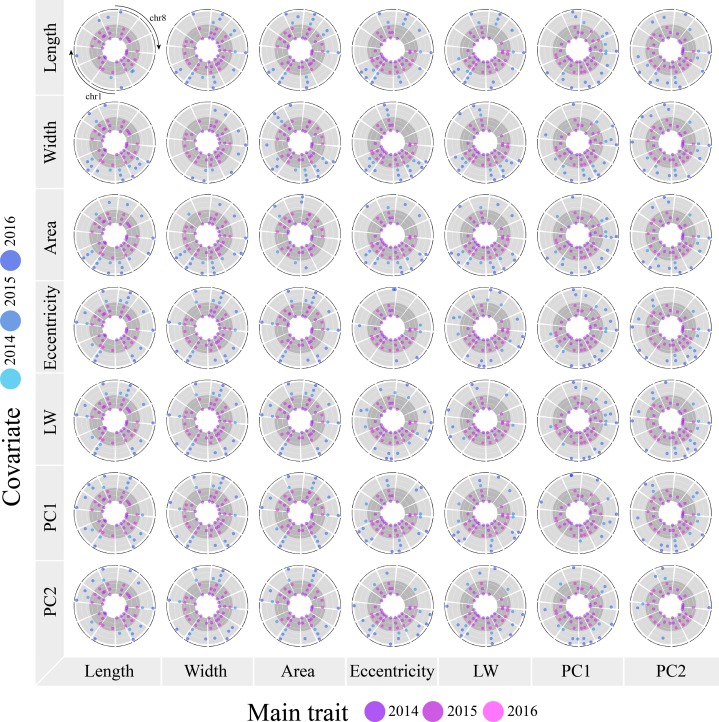
Using covariates for QTL mapping in cranberry fruit quality traits. Columns show the QTL discovered with (blue colored circles, outer three rings in lighter gray background) and without (pink colored circles, inner three rings in darker gray background) the use covariates (rows). Column wise, the inner rings (univariate method, pink circles) are identical since they represent the QTL identified without the use of covariates (serve as reference to identify novel QTL that appear with the addition of covariates).

To partially dissect the confounded QTL effect of correlated traits, we ran *stepwiseQTL* using covariates in a complete diallelic fashion only recording the QTL found consistently in every in all 3 years. In total, we found 44 QTL (collocated in all 3 years) covering all traits (Fig. 4). Of these, 31 were identified previously using the univariate method and 13 were uniquely identified with the use of covariates. As examples, using the univariate method on the trait length, we identified a single QTL in LG4 69.4 cM (explained variance of 4.301%); however, by using covariates (width, area, eccentricity, and LW), we discovered six additional QTL, from which three represented unique novel positions (in LG1 106.1 M, LG2 55.2 cM and LG12 47.2 cM). For width, we found two new QTL, but they corresponded to the same location as the those found in LGs 2 and 12 for the trait length. Similar results were found for area in which the QTL in LG12 was confirmed. For eccentricity and LW, a novel QTL was found in LG7 49.7 cM when using length as covariate; however, similar associations were previously identified in this region for area and width.

## Discussion

In the past few decades, cranberry research has focused mainly on characterizing fruit metabolites with important human health benefits (Cansfield & Francis, 1970; Sapers & Jones, 1986; Sapers & Hargrave, 1987; Vvedenskaya & Vorsa, 2004; Vorsa & Polashock, 2005; Valentova et al., 2007; Su, Howell & D’Souza, 2010; Feldman & Grenier, 2012; Sun et al., 2015), cataloging the genetic diversity of released cultivars and wild germplasm (Vorsa, 2003; Debnath, 2006; Fajardo et al., 2012; Schlautman et al., 2016), and describing the cranberry genome architecture (Fajardo et al., 2013, 2014; Polashock et al., 2014). More recently, previously developed genomic tools have allowed the discovery of several QTL governing important horticultural traits for cranberry growers, including total yield, fruit weight, and fruit rot resistance (Georgi et al., 2013; Schlautman et al., 2015a; Daverdin et al., 2017). However, fruit size and shape, which are emerging as important quality traits used by the cranberry industry, have not been studied. Traditionally, breeders and geneticists have often measured these fruit attributes quantitatively (using time-consuming manual caliper-based methods) or qualitatively (grouping fruit into three major shape categories—round, elongated, and bell-shaped).

### Application of image-based methods in cranberry

Novel image-based phenotyping strategies are being developed for many crops to rapidly collect accurate quantitative data related to fruit size and shape that can be used in breeding, QTL mapping and association studies (Costa et al., 2011). In this study, we implemented a high-throughput phenotyping approach that dramatically increased the amount of fruit measured (per unit of time compared to manual caliper-based measurements). With the imaging setup used here (which involves minimal equipment), we found that a picture of at least 25 fruit can be taken in ∼30 s, which translates into more than 3,000 fruit per hour under well standardized conditions. Moreover, this method provides more accurate data for estimating breeding values for selection and mapping studies. In addition to rapidly capture basic descriptors related to size (length, width, projected area) and shape (LW ratio and eccentricity), we applied a persistent homology algorithm to produce more comprehensive measurements of fruit shape.

Persistent homology has been proven to be a powerful mathematical framework to quantify highly diverse plant morphologies such as leaf shapes, branching topology, and root systems (Chitwood et al., 2016; Klein et al., 2017; Li et al., 2017b, 2018). The main advantages of this method are its ability to process unaligned objects (i.e., orientation-invariant) and to accommodate the different scales commonly found in plant structures (Li et al., 2018). Although the full potential of persistent homology is best observed when applied to highly diverse structures, it has also been used to study phenotypic variation in simpler shapes (Migicovsky et al., 2017). We decided to implement persistent homology in this study for two main reasons. First, cranberry fruit exhibit three main shapes: round, elongated (symmetrical in both main axes), and bell-shaped (symmetrical only across the latitudinal axis). Even though LW ratio discriminates well round from elongated fruit, it does not fully separate elongated from bell-shaped fruit, which can be better studied using methods such as persistent homology. Second, in order to accelerate the imaging process, cranberry fruit in our study were scattered on a white background in a non-defined order or orientation with the only requirement that they were not touching each other. Thus, persistent homology was an excellent alternative to deal with bell-shaped fruit and unaligned fruit objects. Since most of the fruit shape variation observed in the GRYG population was a continuum between round and elongated shapes, persistent homology (at least in the first two PCs) yielded similar results compared with basic descriptors such as LW ratio and eccentricity. Although the main source of fruit shape phenotypic variation in our study fell into the round-to-elongated range, we observed that bell-shaped fruit produced different persistent homology patterns (Fig. S1).

### QTL mapping with image-based data

We performed QTL mapping for the five basic shape and size descriptors and the two persistent homology traits (PC1 and PC2) collected from cranberry fruit images. In total, we found more than 252 QTL for the 21 trait-year combinations of data; from these, more than 40% collocalized for the same trait in at least 2 of the 3 years and provided confidence the identified regions are involved in cranberry fruit shape and size expression. The remaining 60% that were unique within a specific trait/year combination may represent false-positives, or could represent genotype-by-environment interactions for the cranberry fruit traits.

Collocalization of QTL from multiple traits also occurred, which was expected considering the high correlations observed among the shape and size related traits (Fig. 2A). To overcome potential confounding effects of these correlations, we performed QTL mapping a second time using covariates in a diallelic fashion for all trait combinations. The covariate approach was an important strategy that revealed genetic regions involved in the expression of cranberry fruit shape and size that were not identified using the standard univariate approach. These QTL are the first insight into the genetic architecture of fruit size and shape and cranberry, and will serve as a foundation for future fine-mapping, association mapping, and marker-assisted selection programs in cranberry.

### Comparison with previous results and candidate genes

To investigate if fruit size, yield, and mean fruit weight (MFW) are genetically controlled by similar QTL, we searched for overlapping 1.5 LOD intervals between the yield related QTL discovered by Schlautman et al. (2016) and the size-related QTL discovered in the present study. In Schlautman et al. (2016), well-supported QTL for MFW were found in LG10 position 40–50 cM, and LG11 positions ∼10 cM and ∼80 cM. In the same study, QTL for yield were identified in LG11 position ∼25 cM, in close proximity to MFW QTL. Here, we did not find QTL for fruit size related parameters close to the MFW QTL in LG10 described by Schlautman et al. (2016). However, for LG11, we found very significant QTL around ∼20 cM and ∼80 cM (see Fig. 3), which was consistent with the reported QTL found by Schlautman et al. (2016) for almost all the traits studied herein. The fact that similar QTL were identified in the same genomic region across all of the seven traits (including size and shape traits) in our study and for MFW and yield in Schlautman et al. (2016) provide strong evidence of QTL clustering for these traits, which could be due to closely linked genes or pleiotropy and may explain also the high correlations between fruit size, shape, and weight observed in the current study (Fig. 2), and may even be related to yield related traits such as in the case of MFW (Schlautman et al., 2016). Previous studies in important horticultural crops have shown collocated QTL for fruit shape, size, weight, and yield. For example, using two backcross-type populations derived from cultivated *Capsicum annuum* and *C. frutescens*, (Rao et al., 2003) identified several clusters containing QTL for fruit weight, length, diameter, and yield. Other traits such as pericarp width was also collocated, mainly because its intrinsic high correlation with yield related traits.

Further studies should investigate and validate the presence of acting genes and regulatory regions at the QTL locations discovered in this study. In this sense, in File S2, we provide a list of gene models, based on Polashock et al. (2014), located across all marker positions within the genetic map used herein, which could be used as starting point in future fine-mapping QTL and validation studies.

### Breeding implications, industry needs, and future studies

The process of domestication in many fruit crops initially focused in fruit size selection with subsequent efforts to select other agronomic and nutritional traits (Rick, 1995). Although limited compared with other commercial fruit crops mainly due to the recent domestication (<200 years ago) of the crop and lack of genetically diverse pools to incorporate novel traits, cranberry breeding has also initially focused on selection for fruit size, color, and high productivity (Vorsa & Johnson-Cicalese, 2012).

The GRGY population was chosen for this study because it has plentiful genotypic data available (Covarrubias-Pazaran et al., 2016; Schlautman et al., 2017). In minor crops with limited resources, such as cranberry, these populations are extremely valuable and represent the best—or only—opportunity for performing QTL mapping studies for multiple traits. The GRYG population was initially created as a breeding population and not necessarily for genetic or phenotypic studies. Both parents {[BGx(BLxNL)]95 and GH1x35} are second-generation selections with relatively good yield, high anthocyanin content, and round fruit. Although most of the progeny had round or oblong fruit similar to their two parents (data not shown), the population still showed valuable fruit shape and size segregation for QTL mapping. Because the fruit shapes of both parents were mostly non-complex and shape variability was limited in the offspring, the persistent homology method implemented in this study showed some redundant results within the basic shape descriptors (LW ratio and eccentricity), indicating that these are the primary source of variation in cranberry fruit shape. However, given its potential, persistent homology will be extremely useful in future QTL or association studies that make use of larger pools of cranberry genetic and phenotypic diversity, especially when incorporating more complex shapes such as bell-shaped fruit.

Given that the population used here was developed for breeding and selection purposes, our measurements can be immediately used as criteria to select for materials of interest for growers and industry. Among the genotypes with potential for fruit size (considering the traits length, width and area), genotypes P111, P148, P182, P194, P257, P275, P3, P330, P68, and P93, showed consistent values across years in the top 10%. Moreover, considering eccentricity and LW ratio (which measure fruit shape in the same direction), genotypes P104, P112, P121, P129, P136, P14 showed very elongated fruit. On other hand, if the goal is to select large and round fruit at the same time and based on all the traits combined, genotypes P116, P176, and P84 were among the most promising (File S2).

To increase consumption and revenue, the cranberry industry has been developing new high value products based on fruit quality and the inherent cranberry nutritional properties. Therefore, fruit quality characteristics such as fruit color, firmness, size, and shape have become ever more important for cranberry breeding programs, particularly for the production of SDC. For example, the industry requires very specific fruit quality properties to allow efficient industrial processing of these type of products. Thus, varieties with high anthocyanin content and high yield coupled with large round and firm fruit are highly desirable for the industry. Besides the increased production, such ideotype is economically valuable for manufacturing SDC because only large and round cranberries make the sorting for the high value SDC processing lines.

## Summary

Commercial cranberry beds consist of very complex systems and infrastructure that provide the required soil, water, and management requirements of cranberry plants. These beds cannot be easily replicated or afforded at typical public agricultural research stations. Moreover, given the requirements for cranberry production, space availability is very limited for cranberry research and breeding programs. The GRYG population is a precious resource for cranberry breeding and genetics studies that is planted in an active commercial bed. Based on our study of this population, we discovered several linkage groups with promising marker-trait association and clustering areas with QTL collocated for multiple traits. Considering the number of QTL detected, the prevalence of QTL clustering, and the number of collocated QTL for multiple related traits, we found that the availability and use of genome-wide distributed markers to compute relationship/kinship matrices—combined with mixed model approaches—was a useful approach for obtaining accurate BLUP (breeding value estimates). This methodology considerably increased our observed genetic correlations among years and the genomic heritability of the evaluated traits. This strategy is useful in both breeding and genetic research purposes to correct for some of the micro-environmental variation not captured by the explicit design terms when suboptimal planting designs are all that is available or the only design possible.

## Supplemental Information

10.7717/peerj.5461/supp-1 Supplemental Information 1 Analysis code.

10.7717/peerj.5461/supp-2 Supplemental Information 2 Dataset.

10.7717/peerj.5461/supp-3 Supplemental Information 3 Bell-shaped fruit produces different persistent homology patterns. Side views of the annulus kernel-isolated density features of three different fruit shapes. For the bell-shaped fruit (left panel), points a and b highlight the differences detected by the persistent homology method.

## Additional Information and Declarations

### Data Availability

The following information was supplied regarding data availability: The raw data are provided in the Supplemental Files.
