# Supplementary figures and images for: Image-based phenotyping for identification of QTL determining fruit shape and size in American cranberry (Vaccinium macrocarpon L.)
Source: PeerJ. 2018 Aug 15;6:e5461. doi: 10.7717/peerj.5461 (PMC6098679; doi:10.7717/peerj.5461)

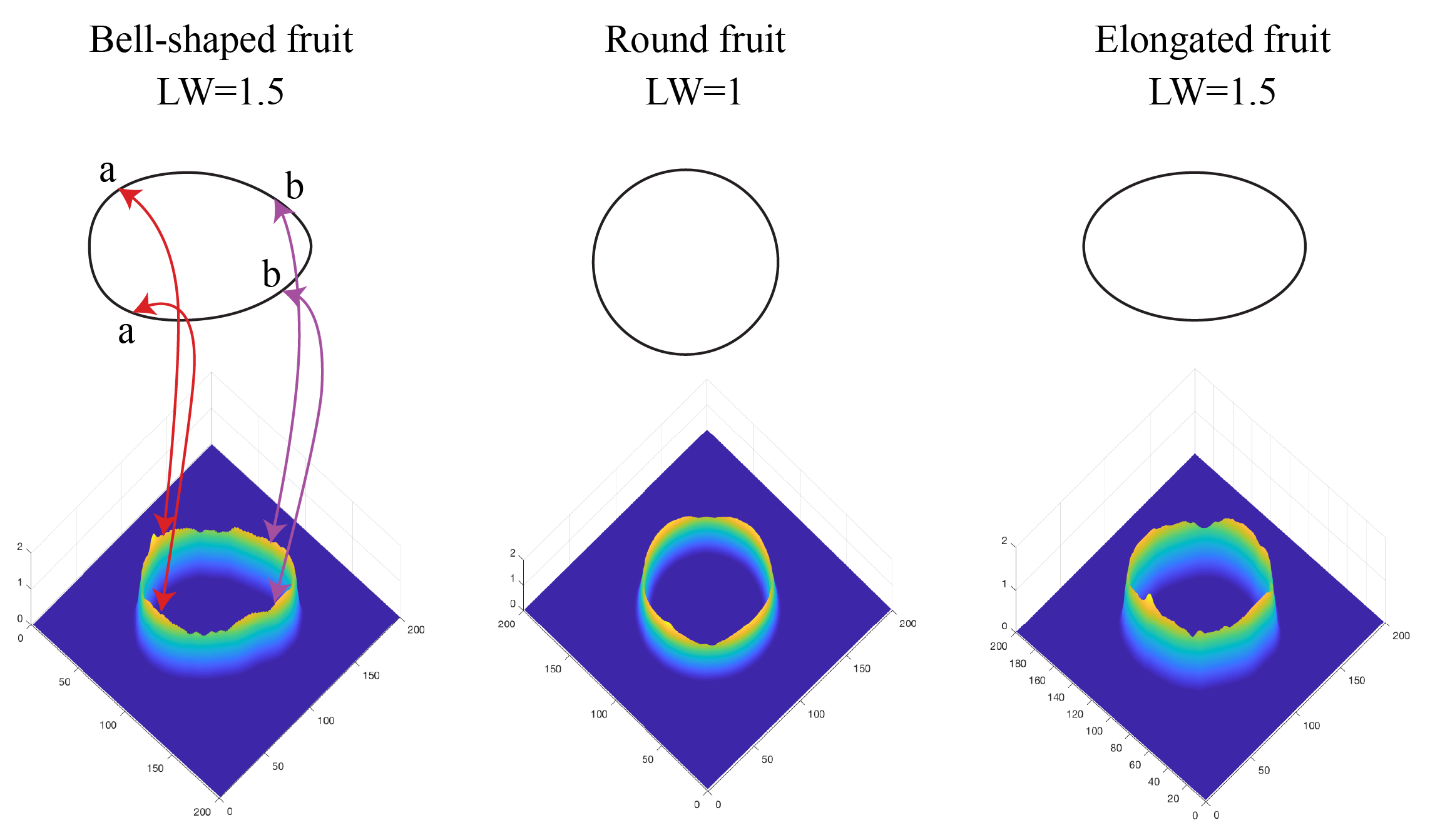

Supplement: Supplemental Information 3 — Side views of the annulus kernel-isolated density features of three different fruit shapes. For the bell-shaped fruit (left panel), points a and b highlight the differences detected by the persistent homology method. [file peerj-06-5461-s003.png]
